# Supplementary material for: Evaluating firefly extinction risk: Initial red list assessments for North America
Source: PLoS One. 2021 Nov 17;16(11):e0259379. doi: 10.1371/journal.pone.0259379 (PMC8598072; doi:10.1371/journal.pone.0259379)
Supplement: S1 File — (DOCX) [file pone.0259379.s006.docx]

# S1 Supporting information

**Detailed IUCN Red List Methodology**

To define a species range, we used the extent of occurrence (EOO), determined by the area of a minimum convex polygon drawn around all known, inferred, or projected sites of present occurrence, and/or the area of occupancy (AOO), based on the area of suitable habitat currently occupied by the species. Taxa qualifying as threatened under Criterion B1 have an extent of occurrence (EOO) of less than 20,000 km^2^. Taxa qualifying as threatened under Criterion B2 have an area of occupancy of less than 2,000 km^2^. According to IUCN guidelines, however, having a restricted range alone is not sufficient to qualify for threatened status under Criterion B. The species in question must also qualify for two of three sub-criteria: a (the taxon is severely fragmented or is found in few locations, which are defined as the area impacted by a specific threat); b (continuing decline has been observed, estimated, inferred, or projected in (i) EOO, (ii) AOO, (iii) area, extent and/or quality of habitat , (iv) number of locations or subpopulations, and/or (v) number of mature individuals; or c (extreme fluctuation in (i) EOO, (ii) AOO, (iii) number of locations for subpopulations, and/or (iv) number of mature individuals) [1].

Taxa meeting the conditions for sub-criterion a are either severely fragmented (“most individuals are found in small and relatively isolated populations”) or occur in less than 10 locations (“a geographically or ecologically distinct area in which a single threatening event can rapidly affect all individuals of the taxon present”). In taxa meeting the conditions for sub-criterion b, continuing decline (“a recent, current, or projected future decline which is liable to continue unless remedial measures are taken”) in (i) EOO, (ii) AOO, (iii) area, extent and/or quality of habitat, (iv) number of locations or subpopulations, and/or (v) the number of mature individuals, has been observed, estimated, inferred, or projected. None of the taxa in this study met the conditions for the third and final sub-criteria; sub-criterion c, which applies to taxa that exhibit extreme fluctuations (“population size or distribution area varies widely, rapidly, and frequently, typically with a variation greater than one order of magnitude”) in (i) EOO, (ii) AOO, (iii) number of locations or subpopulations and/or (iv) the number of mature individuals. For additional details on applying the categories and criteria, refer to the *Guidelines for Using the IUCN Red List Categories and Criteria* [1].

As abundance tends to be correlated with the size of the distribution of a species [2], in some cases population reductions can be suspected from observed reductions in the EOO or AOO of a species or known amounts of habitat loss. However, with North American fireflies, range reductions were difficult to quantify because the available distribution data did not allow for comparison between historical and current range extents. Detailed information on suitable habitats and qualitative data on habitat loss were also scarce. In most cases, locality data was only available for one point in time, often more than thirty years in the past, and few assumptions could be made about the likelihood that occurrence localities remain intact.

The lack of current distribution information was also problematic when calculating EOO and AOO. When only historical records were available, there was high uncertainty in EOO and AOO. This was especially true in regions with limited survey effort. We considered species presence to be uncertain for any occurrence record more than 20 years old. When historical and recent data were available, an upper and lower bound were calculated for the range size parameters. The lower bound encompassed the range where the species was known to be extant and occurrence has been verified in the last 20 years, and the upper bound included all known occurrences, historical and current. Using this method likely improved the accuracy of our species distribution data compared to that used by Reed et al. [3].

Extent of occurrence was calculated for most species. Exceptions included cases where no recent records exist or for Data Deficient species with extremely high uncertainty in distribution limits, for example. However, AOO was only calculated when there was a high level of certainty in the number of occurrence localities, where limited suitable habitat remains, or where sampling effort was extensive enough to rule out occurrences elsewhere. Unless we had evidence to the contrary, occurrence localities were assumed to fit into one 2 x 2 km grid cell (the standard spatial scale used by the IUCN to estimate AOO), as firefly species generally have limited dispersal abilities and tend to aggregate in suitable habitats which are often limited in scale (L. Faust pers. obs.). Area of occupancy was also expressed as a range to reflect recently surveyed occurrences as well as historical occurrences where presence has not been verified recently.

The range maps used to calculate EOO and AOO were created in ArcMap (v. 10.8). These maps are included in the Red List supporting information for each species assessment. There were several mapping protocols followed depending on the data available for each taxon. For widespread species with numerous recent occurrences, distributions were represented with points, as well as polygons encircling those points. In cases where large numbers of occurrence data were not georeferenced, the distributions were represented only with polygons. For habitat specialists with few occurrences, polygons buffered to the estimated dispersal distance for the taxon were draw around each occurrence. Historical occurrences were coded as presence uncertain (since no observations at that locality had been made in 20+ years), and recent occurrences (those reported in the last 20 years) were coded as extant. In these cases, if additional unknown occurrences within the known range of the species were likely, for example where un-surveyed suitable habitat exists, a polygon coded as possibly extant was drawn around all polygon circles representing extant occurrences.

The dispersal capability of adult fireflies is not well known, though in cases where it has been studied and observed, it is evidently limited (L. Faust pers. obs., S. Lewis pers. obs., C. Heckscher pers. obs., L. Buschman pers. comm. 2020). This is especially true for species with flightless females, which likely do not move far beyond their natal habitat [4,5]. Therefore, conservative estimates of dispersal distance were used to buffer polygons, where necessary. Predatory *Photuris* species with relatively mobile females are strong fliers, so maximum dispersal distance was considered to be 10 km. All other species were assigned a dispersal distance of 1 km. Although males may travel farther than 1 km to find a mate, even winged females are relatively poor fliers, especially when they are gravid, and likely do not travel more than a couple hundred meters (L. Buschman pers. comm. 2020). Therefore, for *Photuris* species, occurrences were buffered to 10 km and all other species were buffered to 1 km.

**Additional conservation actions**

**Fill data gaps**

Although the DD category is not a threatened category, we suspect that many species in this group will eventually be assessed as threatened. As such, addressing data gaps for these species will be an important element of a successful firefly conservation strategy. Such species often get overlooked in conservation planning efforts because resources are limited and conservation planners may prioritize species for which more information is available [6]. To address this issue, several authors have made suggestions for prioritizing “potentially threatened” DD species [e.g., 7] or using new methods to assign categories to DD species based on factors such as time elapsed since species description and current species distribution size [8], among others. Based on the scope of our data, we followed Jarić et al. [7] and assigned a “Potentially Threatened” flag to a subset of DD species to help prioritize conservation planning (S5 Table). Species assigned to this subcategory were selected using the following parameters:

1. Species that share extinction risk traits with threatened species (e.g., flightless females, habitat specialization, narrow geographic range, nocturnal courtship activity)
2. Widespread species that have not been reported very much recently or where declines have been reported over small portions of the range
3. Species that have not been reported recently (e.g., the last 20 years), but for which sampling efforts are lacking

**Address taxonomic issues**

Seven species were categorized as DD due to taxonomic uncertainty, highlighting the need for taxonomic revisions of some of the most problematic groups. For example, some species such as *Pyractomena dispersa* and *Ellychnia corrusca* are referred to as species complexes, likely representing multiple undescribed species, while others, like *Photuris pennsylvanica* (which is a misspelling of *P. pensylvanica*), were previously a catch-all for a number of morphologically similar species that have since been split into multiple species [9]. *Ellychnia* and *Photuris* are especially difficult genera, the former needing a revisionary overhaul and the latter comprising many nearly identical looking species that can only be differentiated with years of experience and by considering flash patterns, habitat, geography, and, increasingly, DNA analysis in the identification process. Further complicating matters, many historic records for fireflies may be mis-identified given current taxonomic delineations, and redeterminations of these specimens are difficult because these records lack the necessary flash pattern data, which was rarely recorded with collections. It is also highly likely that fireflies are under-reported from certain areas, particularly in western North America, whether due to limited interest where flashing fireflies are less widespread, smaller human populations, or because species are cryptic or rare. Targeted surveys for taxonomically complicated species, coupled with careful examination of associated geography, seasonality, and flash pattern (where applicable) may help with delineations, but the use of DNA barcoding and other genetic tools should also be considered to complement more traditional morphological studies.

**References**

1. IUCN Standards and Petitions Committee. Guidelines for using the IUCN Red List Categories and Criteria. Version 14. Prepared by the Standards and Petitions Committee. 2019. Available: http://www.iucnredlist.org/documents/RedListGuidelines.pdf

2. Holt AR, Gaston KJ, He F. Occupancy-abundance relationships and spatial distribution: A review. Basic and Applied Ecology. 2002;3: 1–13. doi:10.1078/1439-1791-00083

3. Reed JM, Nguyen A, Owens ACS, Lewis SM. Linking the seven forms of rarity to extinction threats and risk factors: an assessment of North American fireflies. Biodivers Conserv. 2020;29: 57–75. doi:10.1007/s10531-019-01869-7

4. Wing SR. *Photinus collustrans*: Reproductive ecology of flightless female fireflies (Coleoptera: Lampyridae). University of Florida. 1988. Available: https://ufdc.ufl.edu/AA00004830/00001

5. Cicero JM. Lek assembly and flash synchrony in the Arizona firefly *Photinus knulli* Green (Coleoptera: Lampyridae). Coleopt Bull. 1983;37: 318–342.

6. Bland LM, Bielby J, Kearney S, Orme CDL, Watson JEM, Collen B. Toward reassessing data-deficient species. Conserv Biol. 2017;31: 531–539. doi:10.1111/cobi.12850

7. Jarić I, Courchamp F, Gessner J, Roberts DL. Potentially threatened: A Data Deficient flag for conservation management. Biodivers Conserv. 2016;25: 1995–2000. doi:10.1007/s10531-016-1164-0

8. Morais AR, Siqueira MN, Lemes P, Maciel NM, De Marco P, Brito D. Unraveling the conservation status of Data Deficient species. Biol Conserv. 2013;166: 98–102. doi:10.1016/j.biocon.2013.06.010

9. Lloyd JE. A naturalist’s long walk among shadows of North American *Photuris*: Patterns, outlines, silhouettes... echoes. Bridgen Press; 2018.
