# Supplementary material for: Evaluating firefly extinction risk: Initial red list assessments for North America
Source: PLoS One. 2021 Nov 17;16(11):e0259379. doi: 10.1371/journal.pone.0259379 (PMC8598072; doi:10.1371/journal.pone.0259379)
Supplement: Attachment — Submitted filename: PONE-D-21-17592_reviewer1.pdf [file pone.0259379.s007.pdf]

PLOS ONE

Evaluating firefly extinction risk: Initial Red List assessments for North America

--Manuscript Draft--

**Manuscript Number:**	PONE-D-21-17592
**Article Type:**	Research Article
**Full Title:**	Evaluating firefly extinction risk: Initial Red List assessments for North America
**Short Title:**	Extinction risk for North American fireflies
**Corresponding Author:**	Candace E. Fallon<br>The Xerces Society for Invertebrate Conservation<br>Portland, OR UNITED STATES
**Keywords:**	Lampyridae; conservation status; extinction risk; IUCN Red List; firefly; invertebrate
**Abstract:**	Fireflies are a family of charismatic beetles known for their bioluminescent signals. Recent anecdotal reports suggest that firefly populations in North America may be in decline. However, prior to this work, no studies have undertaken a systematic compilation of geographic distribution, habitat specificity, and threats facing North American fireflies. To better understand their extinction risks, we conducted baseline assessments according to the categories and criteria of the International Union for Conservation of Nature (IUCN) Red List for 132 species from the United States and Canada (approximately 79% of described species in the region). We found at least 18 species (14%) to be under threat of extinction from various threats, including habitat loss, light pollution, and climate change (sea level rise and drought). In addition, more than half of the species (53%) could not be assessed due to insufficient data, highlighting the need for further study. Future research and conservation efforts should prioritize monitoring and protecting populations of at-risk species, preserving and restoring habitat, gathering data on population trends, and filling critical information gaps for data deficient species suspected to be at risk.
**Order of Authors:**	Candace E. Fallon
	Anna Walker
	Sara Lewis
	Joseph Cicero
	Lynn Faust
	Christopher M. Heckscher
	Cisteil X. Pérez-Hernández
	Ben Pfeiffer
	Sarina Jepsen
**Additional Information:**	
**Question**	**Response**
**Financial Disclosure**<br><br>Enter a financial disclosure statement that describes the sources of funding for the work included in this submission. Review the submission guidelines for detailed requirements. View published research articles from *PLOS ONE* for specific examples.<br><br>This statement is required for submission	CEF and SJ were funded by the Samuel Freeman Charitable Trust, the Edward Gorey Charitable Trust, the New-Land Foundation (http://newlandfoundation.org/), Morningstar Foundation (http://themorningstarfoundation.com/), and Xerces Society members. AW was funded by the New Mexico BioPark Society (https://www.bioparksociety.org/main/). The funders had no role in study design, data collection and analysis, decision to publish, or preparation of the manuscript.

Powered by Editorial Manager® and ProduXion Manager® from Aries Systems Corporation

and **will appear in the published article** if the submission is accepted. Please make sure it is accurate.<br><br>**Unfunded studies**<br>Enter: *The author(s) received no specific funding for this work.*<br><br>**Funded studies**<br>Enter a statement with the following details:<br>• Initials of the authors who received each award<br>• Grant numbers awarded to each author<br>• The full name of each funder<br>• URL of each funder website<br>• Did the sponsors or funders play any role in the study design, data collection and analysis, decision to publish, or preparation of the manuscript?<br>• **NO** - Include this sentence at the end of your statement: *The funders had no role in study design, data collection and analysis, decision to publish, or preparation of the manuscript.*<br>• **YES** - Specify the role(s) played.<br><br>* typeset	
**Competing Interests**<br><br>Use the instructions below to enter a competing interest statement for this submission. On behalf of all authors, disclose any competing interests that could be perceived to bias this work—acknowledging all financial support and any other relevant financial or non-financial competing interests.<br><br>This statement is required for submission and **will appear in the published article** if the submission is accepted. Please make sure it is accurate and that any funding sources listed in your Funding Information later in the submission form are also declared in your Financial Disclosure statement.<br><br>View published research articles from *PLOS ONE* for specific examples.	The authors have declared that no competing interests exist.

Powered by Editorial Manager® and ProduXion Manager® from Aries Systems Corporation

**NO authors have competing interests**<br><br>Enter: *The authors have declared that no competing interests exist.*<br><br>**Authors with competing interests**<br><br>Enter competing interest details beginning with this statement:<br><br>*I have read the journal's policy and the authors of this manuscript have the following competing interests: [insert competing interests here]*<br><br>* typeset	
**Ethics Statement**<br><br>Enter an ethics statement for this submission. This statement is required if the study involved:<br><br>• Human participants<br>• Human specimens or tissue<br>• Vertebrate animals or cephalopods<br>• Vertebrate embryos or tissues<br>• Field research<br><br>Write "N/A" if the submission does not require an ethics statement.<br><br>General guidance is provided below. Consult the submission guidelines for detailed instructions. **Make sure that all information entered here is included in the Methods section of the manuscript.**	N/A

Powered by Editorial Manager® and ProduXion Manager® from Aries Systems Corporation

**Format for specific study types**<br><br>**Human Subject Research (involving human participants and/or tissue)**<br>• Give the name of the institutional review board or ethics committee that approved the study<br>• Include the approval number and/or a statement indicating approval of this research<br>• Indicate the form of consent obtained (written/oral) or the reason that consent was not obtained (e.g. the data were analyzed anonymously)<br><br>**Animal Research (involving vertebrate animals, embryos or tissues)**<br>• Provide the name of the Institutional Animal Care and Use Committee (IACUC) or other relevant ethics board that reviewed the study protocol, and indicate whether they approved this research or granted a formal waiver of ethical approval<br>• Include an approval number if one was obtained<br>• If the study involved *non-human primates*, add *additional details* about animal welfare and steps taken to ameliorate suffering<br>• If anesthesia, euthanasia, or any kind of animal sacrifice is part of the study, include briefly which substances and/or methods were applied<br><br>**Field Research**<br><br>Include the following details if this study involves the collection of plant, animal, or other materials from a natural setting:<br>• Field permit number<br>• Name of the institution or relevant body that granted permission	
**Data Availability**<br><br>Authors are required to make all data underlying the findings described fully available, without restriction, and from the time of publication. PLOS allows rare exceptions to address legal and ethical concerns. See the PLOS Data Policy and FAQ for detailed information.	Yes - all data are fully available without restriction

A Data Availability Statement describing where the data can be found is required at submission. Your answers to this question constitute the Data Availability Statement and **will be published in the article**, if accepted.<br><br>**Important:** Stating 'data available on request from the author' is not sufficient. If your data are only available upon request, select 'No' for the first question and explain your exceptional situation in the text box.<br><br>Do the authors confirm that all data underlying the findings described in their manuscript are fully available without restriction?	
**Describe where the data may be found in full sentences. If you are copying our sample text, replace any instances of XXX with the appropriate details.**<br><br>• If the data are **held or will be held in a public repository**, include URLs, accession numbers or DOIs. If this information will only be available after acceptance, indicate this by ticking the box below. For example: *All XXX files are available from the XXX database (accession number(s) XXX, XXX.).*<br>• If the data are all contained **within the manuscript and/or Supporting Information files**, enter the following: *All relevant data are within the manuscript and its Supporting Information files.*<br>• If neither of these applies but you are able to provide **details of access elsewhere**, with or without limitations, please do so. For example:<br><br>*Data cannot be shared publicly because of [XXX]. Data are available from the XXX Institutional Data Access / Ethics Committee (contact via XXX) for researchers who meet the criteria for access to confidential data.*<br><br>*The data underlying the results presented in the study are available from (include the name of the third party*	All relevant data are within the manuscript and its Supporting Information files

and contact information or URL).<br>• This text is appropriate if the data are owned by a third party and authors do not have permission to share the data.<br><br>* typeset	
Additional data availability information:	

Manuscript
Click here to access/download;Manuscript;Evaluating firefly extinction risk_2021-05-27_final.docx

Evaluating firefly extinction risk: Initial Red List assessments for North America

Candace E. Fallon[1,9*], Anna Walker[2,9], Sara Lewis[3,9], Joseph Cicero[4], Lynn Faust[5,9], Christopher M. Heckscher[6,9], Cisteil X. Pérez-Hernández[7,9], Ben Pfeiffer[8,9], and Sarina Jepsen[1,9]

[1]The Xerces Society for Invertebrate Conservation, Portland, Oregon, United States of America

[2]New Mexico BioPark Society, Albuquerque, New Mexico, United States of America

[3]Department of Biology, Tufts University, Medford, Massachusetts, United States of America

[4] School of Plant Sciences, University of Arizona, Tucson, AZ, United States of America

[5]Emory River Land Co., Knoxville, Tennessee, United States of America

[6]Department of Agriculture and Natural Resources, Delaware State University, Dover, Delaware, United States of America

[7]Instituto de Investigaciones en Ecosistemas y Sustentabilidad, Universidad Nacional Autónoma de México, Michoacán, México

[8]Firefly Conservation and Research, New Braunfels, Texas, United States of America

[9]IUCN SSC Firefly Specialist Group, Gland, Switzerland

[*]Corresponding author

E-mail: candace.fallon@xerces.org

Abstract

Fireflies are a family of charismatic beetles known for their bioluminescent signals. Recent anecdotal reports suggest that firefly populations in North America may be in decline. However, prior to this work, no studies have undertaken a systematic compilation of geographic distribution, habitat specificity, and threats facing North American fireflies. To better understand their extinction risks, we conducted baseline assessments according to the categories and criteria of the International Union for Conservation of Nature (IUCN) Red List for 132 species from the United States and Canada (approximately 79% of described species in the region). We found at least 18 species (14%) to be under threat of extinction from various threats, including habitat loss, light pollution, and climate change (sea level rise and drought). In addition, more than half of the species (53%) could not be assessed due to insufficient data, highlighting the need for further study. Future research and conservation efforts should prioritize monitoring and protecting populations of at-risk species, preserving and restoring habitat, gathering data on population trends, and filling critical information gaps for data deficient species suspected to be at risk.

Introduction

Effective conservation planning and action depends on identifying the most at-risk species based on their estimated probability of extinction. The International Union for Conservation of Nature (IUCN) Red List of Threatened Species is considered the global standard for estimating the risk of species extinction and can be used as a first step in conservation efforts [1,2]. First established in 1964, major gains have been made in adding new assessments to the Red List in recent years, moving ever closer to the group's goal of 160,000 assessed species. Currently, the Red List comprehensively covers charismatic vertebrates, including mammals (91% of all species assessed) and birds (100% of species assessed) [3].

Invertebrates, in contrast, are profoundly underrepresented on the Red List, with just 2% of described species (24,219 out of an estimated 1,478,938) assessed as of 2020 [3]. This gap is even wider for insects: although these represent an estimated 53% of described animal and plant species, only 1% of these have been assessed [3].

Beetles, a hyper-diverse group of insects with an estimated 386,500 described extant species worldwide [4] have been identified as a priority group for Red Listing due to their species richness, assessment practicality (e.g., relatively stable taxonomy, adequate information available), and economic value [5]. The firefly beetles (family Lampyridae), which contain some 2,200 species globally [4], represent an ideal group for Red List assessments because these charismatic and cosmopolitan insects have the potential to serve as flagship species for invertebrate conservation. They possess diverse life history traits and behaviors and have been the subject of active evolutionary, behavioral, and genetic research [6–10]. Firefly luciferase has facilitated numerous scientific advances in biomedicine [e.g., 11]. Furthermore, fireflies are culturally, ecologically, and economically important, and because of their sensitivity to light pollution and other environmental degradation, they may be important bioindicators of ecosystem health [12–17]. Some species have been used as biological control agents of unwanted land snails [18].

Long-term surveys have revealed local population declines of the glow-worm *Lampyris noctiluca* in the U.K. [19,20] and the congregating mangrove firefly *Pteroptyx tener* in Malaysia [21,22]. In North America, population declines have been anecdotally reported [16], but IUCN Red List assessments had yet to be conducted for any firefly species. A recent review of global threats to firefly persistence revealed that habitat degradation and loss, light pollution, pesticide use, poor water quality, climate change, and invasive species to be among the major suspected drivers of decline [23]. Firefly tourism, which has increased rapidly in recent years and has been identified as a potential threat, offers an

opportunity to examine how human activities can affect fireflies and their habitats, while determining how these activities can continue without causing local extirpations [17]. With emerging evidence for widespread declines in insect populations [24–26], there is an urgent need for formal assessments to inform the conservation status of firefly species and estimate their extinction risk.

This study summarizes global IUCN Red List assessments for fireflies in the U.S. and Canada, presenting the first formal estimates of extinction risk conducted for any member of this beetle family. We compiled available information on distributions, habitats, life history traits, behaviors, and threats for most (79%) of the currently described firefly species in the U.S. and Canada. Our goal in compiling this baseline data was to identify species at greatest risk of extinction, propose strategies for conserving threatened species, and highlight targets for future research.

Methods

Study organism

Fireflies (Coleoptera: Lampyridae) are holometabolous insects that spend the majority of their lives as larvae – sometimes up to 2 years or more – whereas adults may live only a few weeks [27]. Generation time and seasonality vary considerably depending on latitude, elevation, degree day range, and sex and species-specific emergence timing, in addition to weather and climate [28]. In general, generation time increases with higher latitudes and elevations. Southern fireflies may have one-year life cycles, whereas northern populations could have two to three-year life cycles [28]. However, because fireflies are facultative in their development time, this period may increase in response to environmental variables such as drought [28] or increases in elevation (L. Buschman pers. comm. 2020).

Similarly, the breeding season may be longer (year-round for some species) at southern latitudes, while it will be much shorter at higher latitudes or elevations (lasting only a week to a few months) [28].

As larvae, fireflies are voracious predators of soft-bodied invertebrates including snails, slugs, and worms [9], but may also be scavengers of dead insects and berries [29]. They are typically subterranean or found on or near the soil surface, in leaf litter, or in rotting logs, depending on the genera and/or species [16,28,30]. Adults of most species are not known to feed, although some species have been observed nectaring on flowers, mouthing leaves, and feeding on sap [28,31–34], and the females of some *Photuris* species are predatory mimics of other fireflies [35,36].

Although fireflies are known for bioluminescence, the actual bioluminescent capabilities of the group as a whole are these: the larvae of all known firefly species are luminescent [9], and not all adults are capable of producing light. In the U.S. and Canada, fireflies can thus be organized into groups based on their bioluminescent capabilities: those that use flashing or glowing courtship signals (flashing fireflies and glow-worms), and those that do not (daytime dark species; in this context, 'dark' refers to non-luminescent or faintly luminescent diurnal species). Flashing fireflies, also known as lightningbugs, are typically crepuscular or nocturnally active; male and female adults use precisely timed flashes or flickers to communicate with potential mates [9]. Glow-worms are active during a similar time period but differ in that adult female glow-worms are typically flightless because their wings are short or even absent [9]. Furthermore, it is primarily the adult females that are luminescent, glowing to attract often non-luminescent males that fly overhead in search of a mate (there are some exceptions to this, e.g. *Phausis reticulata*) [9,37]. Daytime dark fireflies are diurnally active and are known [38] or suspected to use pheromones to locate potential mates [6,8].

Fireflies require moist conditions to prevent desiccation of larvae and their prey [9,16]. In general, fireflies are found in diverse habitats, including riparian woodlands, deserts, and coastal salt

marshes. While some species are strict habitat specialists, others utilize a variety of habitats. Certain species opportunistically occupy urban and rural areas such as residential lawns, crop fields, and overgrown lots.

Species checklist

We compiled a checklist of all native described species and subspecies of Lampyridae found in the U.S. and Canada based on Lloyd [39], which we updated to include recent species descriptions [30,40–42]. This yielded 167 species in 20 genera (S1 Table). Thirty-nine of these species were described in just the last 15 years [30,40–42], supporting speculation that as many as 225 species could occur in the U.S. and Canada [9]. One introduced European species, *Phosphaenus hemipterus*, reported from Nova Scotia [43], was not included. Synonymy was addressed using ITIS [44], Cicero [45], and other taxonomic references, where relevant. The updated checklist was reviewed by firefly experts (S2 Table). Thirty-five recently described *Photuris* species [30] were excluded due to a paucity of data and lack of knowledgeable taxonomic experts, yielding a total of 1[illegible] species that were assessed.

Literature review and data compilation

At the outset of the assessment process, we reviewed published literature and unpublished reports and solicited input from taxonomic experts. For 130 species and two subspecies, we compiled information on taxonomy, distribution, population size, ecology, behavior, threats, and any known conservation measures. Occurrence records were obtained from online biodiversity databases and museum collections (e.g., GBIF, SCAN, California Academy of Sciences), scientific literature, and species experts. Data were screened for anomalous records, which were vetted and removed if questionable. Unless pertaining to widespread or common species, observations from iNaturalist and BugGuide

community science sites were only used if they had been verified by a taxonomic expert (Role=Determiner, see S2 Table). In some cases, records from the published literature were georeferenced in order to draft more detailed distribution maps.

IUCN Red List methodology

We evaluated extinction risk for each species using the IUCN Red List Categories and Criteria: Version 3.1 [46]. Each species was assessed against five criteria with quantitative thresholds, which are based on standard biological indicators that render populations more vulnerable to extinction: A (past, present, or future population size reduction), B (geographical range size with evidence of decline, fragmentation, or fluctuation), C (small population size with decline, fragmentation, or fluctuation), D (very small or restricted population), and E (quantitative analysis of extinction risk).

Depending on which criteria thresholds were met, each taxon was assigned to one of the following IUCN Red List categories: Extinct (EX), Extinct in the Wild (EW), Critically Endangered (CR), Endangered (EN), Vulnerable (VU), Near Threatened (NT), Least Concern (LC) or Data Deficient (DD). Species assigned to the categories CR, EN, or VU are considered threatened because they are facing extremely high, very high, or high risk of extinction in the wild, respectively. Species assessed as Near Threatened are close to qualifying for a threatened category and therefore may qualify as threatened in the near future. Species assessed as Least Concern are generally widespread and abundant and do not qualify for a threatened category under any of the criteria. A taxon is considered Data Deficient when there is not enough information on the distribution or population size to make a direct or indirect assessment of its extinction risk. Species are assessed as Extinct when there is no reasonable doubt that the last individual has died, and species are assessed as Extinct in the Wild when they are known to survive onl[illegible]ptivity [47]. Like many invertebrates, none of the species assessed had sufficient

information on population size or rates of population size reduction to be evaluated against the thresholds for Criteria A, C, D and E. Therefore, all species assessed as threatened were done so under Criterion B, which is based on restricted ranges with evidence of decline, fragmentation, or fluctuation. Further details on the Red List methodology can be found in S1 Protocol.

Synthesis and review

Throughout the process, species experts (S2 Table) were consulted to verify that each species assessment and distribution map included accurate and up-to-date information. The majority of assessments (128 species) were published on the IUCN Red List in March 2021 [48], while the remaining four species are awaiting publication.

Results and discussion

Species distributions

Fireflies were recorded in every U.S. state except for Hawaii and every Canadian province and territory except Nunavut (Fig 1A; S1 Table). Thirty species (23%) were thought to be endemic to a single state or province (27% of which are categorized as threatened). States that support the highest numbers of endemic species include Arizona (eight species), Florida (eight species), California (five species), and Texas (four species) (S1 Table). In general, species richness increases moving from west to east; when we overlaid Level III Ecoregion [49] boundaries on the map, the major hotspots of species richness (defined here as areas with more than 30 species across most of the ecoregion) were in the North Central Appalachians, Northern Allegheny Plateau, Northern Piedmont, and Blue Ridge ecoregions (Fig 1A). The Middle Atlantic Coastal Plain and Southeastern Plains also support more than 30 species each,

but only across a small part of the ecoregion. Threatened species are concentrated in the Mid-Atlantic and Southeast regions (Fig 1B), while DD species are scattered throughout the two countries (Fig 1C). All 18 threatened species have narrow geographic ranges, with 10 thought to be endemic to a single state. It is likely that these distributions are heavily influenced by sampling bias and geographic concentrations of species experts; for example, West Virginia likely has higher species richness than is currently reported (12 species) given the high number of species found in surrounding states, but sampling efforts are not yet as comprehensive in this state.

Fig 1. Species distributions and status summaries. (A) Overall species richness of fireflies in the U.S. and Canada. Dark lines indicate Level III Ecoregion boundaries. (B) Geographic summary of threatened (CR, EN, or VU) firefly species. Note that the 2 species indicated in Arizona include 2 subspecies. (C) Geographic summary of data deficient firefly species (as a percent of the total number of species reported from each state).

Extinction risk and threats

Our assessments suggest that at least 14% of evaluated North American firefly species (18 species) are threatened, classified as either Critically Endangered (CR)[illegible] Endangered ([illegible] or Vulnerable (VU)[illegible]Table 1). In addition, 12%[illegible]were categorized as Near Threatened (NT) and 32% were classified as Least Concern (L[illegible]ver half (53[illegible]of assessed firefly species were categorized as Data Deficient (DD), which means there remains considerable uncertainty in the proportion of North American fireflies that may be at risk of extinction. Our estimate of 14% threatened likely represents a lower limit, with an upper limit of 67% should all DD species turn out to be threatened. Following methods used in other Red List assessments [50,51], if we assume that our da[illegible]D[illegible]eficient species follow a pattern similar to

those with sufficient data, we estimate that 29% (a “mid-estimate”) of North American firefly species may eventually be classified as threatened.

Table 1: Conservation status for 132 North American firefly species

IUCN Red List Category	Count	Percentage
Extinct (EX)	0	0%
Extinct in the Wild (EW)	0	0%
Critically Endangered (CR)	1	1%
Endangered (EN)	10	8%
Vulnerable (VU)	7	5%
Near Threatened (NT)	2	2%
Least Concern (LC)	42	32%
Data Deficient (DD)	70	53%
**Summary information**		
Total species evaluated	132	
Total species with sufficient data (CR+EN+VU+NT+LC)	62	47%
Total Threatened - lower limit estimate (CR+EN+VU)	18	14%
Total Threatened – mid estimate ((CR+EN+VU)/(total - DD)*total)	38	29%

Total Threatened - upper limit estimate (CR+EN+VU+DD)	88	67%

Invertebrate extinction risk has been linked to several different factors, including narrow geographical ranges, habitat specialization, and body size [52–54]. For fireflies, Reed et al. [55] identified risk factors expected to make species more susceptible to threats, including courtship activity period (nocturnal vs. diurnal), poor dispersal ability (due in part to adult female brachyptery or aptery), and habitat specialization. In our assessments, these risk factors were found to be prevalent among firefly species with heightened extinction risk (Table 2).

Table 2: Ecology and life history characteristics of 18 threatened firefly species in the U.S. and Canada.

Species name	Common name	Category	Criteria
Bicellonycha wickershamorum	Southwest spring firefly	VU	B1ab(iii)
Bicellonycha wickershamorum piceum	Gila Southwest spring firefly	EN	B2ab(iii)
Bicellonycha wickershamorum wickershamorum	Southwest spring firefly	VU	B1ab(iii)
Lucidota luteicollis	Florida scrub dark firefly	VU	B1ab(iii)
Micronaspis floridana	Florida intertidal firefly	EN	B2ab(i,ii,iii)
Photinus acuminatus	Pointy-lobed firefly	EN	B2ab(i,ii,iii,iv,v)
Photinus knulli	Southwest synchronous firefly	VU	B1ab(iii)
Photuris bethaniensis	Bethany Beach firefly	CR	B1ab(i,ii,iii,v)
Photuris cinctipennis	Belted firefly	EN	B1ab(ii,iii)+2ab(ii,iii)
Photuris flavicollis	Sky island firefly	VU	B1ab(iii)
Photuris forresti	Loopy five firefly	EN	B1ab(i,ii,iii,iv)+2ab(i,ii,iii,iv
Photuris mysticalampas	Mysterious lantern firefly	EN	B1B2ab(ii,iii)
Photuris pensylvanica	Dot-dash firefly	VU	B2ab(iii)
Photuris pyralomima	None	EN	B1ab(i,ii,iii)+B2ab(i,ii,iii)
Photuris walldoxeyi	Cypress firefly	VU	B2ab(iii)
Pleotomodes needhami	Ant-loving scrub firefly	EN	B1ab(iii)
Pyractomena ecostata	Keel-necked firefly	EN	B2ab(i,ii,iii)

Pyractomena vexillaria	Amber comet	EN	B2ab(i,iii)

For species with sufficient information to identify known and suspected threats to their persistence (88 species total), the primary threats included habitat loss and degradation, light pollution, and climate change and severe weather. Habitat loss has been identified as the biggest perceived threat to fireflies worldwide [23], rendering habitat specialists particularly vulnerable. All 18 species categorized as threatened are known or suspected to be restricted to specialized habitats like freshwater interdunal swales or cypress swamps (Fig 2), which makes them more vulnerable to habitat loss, degradation, and fragmentation. These threats are caused by a variety of human activities, including commercial and residential development, agricultural conversion, water pollution, groundwater pumping, waterway modifications, cattle grazing, and recreational activities such as off-road vehicle (ORV) use. Habitat loss and degradation can be particularly devastating for species with flightless females, which are more vulnerable to trampling or habitat destruction due to their limited dispersal capacity. Two of the species categorized as threatened, the Florida scrub dark firefly (*Lucidota luteicollis*) and the ant-loving scrub firefly (*Pleotomodes needhami*), have flightless adult females. Considering that approximately a quarter of firefly species in the U.S. and Canada have or are expected to have flightless females, 23 species (68%) of which were categorized as DD, it is likely that additional species will eventually be categorized as threatened.

Fig 2. Threatened fireflies tend to be restricted to specialized habitats. From top: *Lucidota luteicollis* and upland sand scrub, FL (L: Brandon Woo, R: Leo Miranda/USFWS); *Micronaspis floridana* and coastal salt marsh, FL (L: Drew Fulton, R: Rain0975); *Photuris bethaniensis* and interdunal swale, DE (L: Christopher M. Heckscher, R: Emily May); *Photuris walldoxeyi* and cypress swamp, MS (L: Luiz Silveira, R: Visit Mississippi); *Pyractomena ecostata* and Atlantic tidal marsh, DE (L: Oliver Keller, R: Andy Atzert); *Pyractomena vexillaria* and habitat along the Devils River, TX (L: Mike Quinn, R: Ben Pfeiffer).

In general, moisture is critically important during all firefly life stages to prevent desiccation [9]; eggs, soft-bodied larvae, and flightless females may be particularly susceptible [56]. Thus, loss of moisture due to habitat manipulation, drought, or mismanagement of water resources can negatively impact fireflies. Because firefly larvae are predatory on soft-bodied invertebrates that are also susceptible to desiccation, loss of moisture can impact prey populations as well. Climate change is likely to be a major concern for many species. In the arid American West, droughts are becoming more widespread, frequent, and severe due to a changing climate [57]. As a result of this, combined with changing precipitation patterns and increasing human demands, water tables are dropping [58,59], which can cause ephemeral aquatic habitats to go dry, interrupt flow regimes, and stress local plant communities [60]. For example, some western firefly habitats have completely disappeared due to water table reductions [61], and continuing declines in plant communities along riparian corridors in Texas are causing reduced moisture retention in the soil, which contributes to lower quality habitat for firefly larvae and diminishes the amount of water available to recharge aquifers (B. Pfeiffer pers. obs.).

Wetland habitats overall are in decline across the U.S., primarily from development; over a 200-year period from the 1780s to 1980s, the contiguous U.S. lost an estimated 53% of original wetlands [62]. More recently, although the pace of loss appears to have slowed [63], wetland loss continues to occur at a high rate in certain regions. For example, the northeast and southeast regions of the U.S., where firefly species richness is highest (Fig 1A), both saw downward trends in wetlands acreage from 1992 to 2010 [64]. Coastal regions are particularly at risk; an estimated 80,000 acres of coastal wetlands in the contiguous U.S. are lost each year due to development, drainage, storms, and sea level rise [63]. Loss of wetland habitat due to sea level rise was identified as a major threat to coastal firefly species like *Photuris bethaniensis* and *Micronaspis floridana*. Because these are habitat specialists and occupy small areas threatened by intense coastal development, their capacity to disperse to other sites is limited [65].

Development is also linked to light pollution, or artificial light at night (ALAN), a threat affecting 17 out of 18 threatened firefly species. ALAN is comprised of skyglow (the diffuse glowing haze over populated areas), glare (excessive amounts of lighting), and light trespass (light that spills out beyond its intended target). It can be caused by a number of different sources, from commercial and residential development to vehicle headlights and gas flares. All sources of ALAN have the potential to drive firefly population declines. More than 75% of firefly species in the United States and Canada are nocturnally active or crepuscular species that utilize bioluminescent courtship signals that are sensitive to environmental light conditions. A growing body of research suggests that artificial light from street lamps, residences, and other sources may overwhelm the optical systems of fireflies and impede the ability of males to locate female mates [23,66]. For example, experimental studies have shown that artificial light can interfere with the production and reception of courtship signals [67,68] and inhibit larval dispersal [69], which could affect reproductive fitness and have cascading impacts for firefly populations.

Moving forward: Conservation actions

The results of our assessments have made it clear that additional conservation actions are needed for fireflies in the U.S. and Canada (S3 Table). More specifically, this includes identifying and protecting populations of at-risk species, preserving and restoring firefly habitat, gathering data on population trends, and filling critical information gaps for data deficient species suspected to be at risk. Science communication is also important: conducting education and outreach can help ensure that fireflies and their needs are taken into consideration. In the following sections, we expand on these recommended next steps to prevent firefly species extinctions.

Protect at-risk species

These assessments identified 18 species at risk of extinction and two others that may be at risk in the near future (Near Threatened). Currently, very few conservation measures are ~~currently~~ in place to protect North American fireflies. Species-specific conservation actions should focus on prioritizing these threatened species. The Critically Endangered Bethany Beach firefly, *Photuris bethaniensis*, which is listed as State Endangered in Delaware, is currently under consideration for Endangered Species Act (ESA) listing—the first firefly to be petitioned [70]. No other fireflies are included in endangered species lists for any state or province, and no regulatory mechanisms are in place to protect at-risk species. However, several states, including Delaware, Florida, Indiana, Maryland, and South Carolina, do include at-risk firefly species as Species of Greatest Conservation Need (SGCN) in their State Wildlife Action Plans. These plans are intended to inform conservation priorities and actions at the state level, with a particular focus on strategies for managing and protecting SGCN. We recommend that state wildlife agencies include threatened firefly species as SGCN, if they are not already included. Furthermore, we suggest that D[illegible]pecies that we suspect to be threatened also be considered as SGCN (S5 Table). In addition to these efforts, we support converting the Red List assessments so that each species can be assessed using the Nature[illegible]ve ranking calculator and made available to the NatureServe Network. This will further enable states to assign state ranks that could streamline conservation and funding efforts for species found within their borders.

Preserve and restore habitat

Because habitat loss is a key threat to fireflies, preserving and restoring habitat for threatened firefly species will be an integral part of any conservation efforts. This can be accomplished in several ways:

- Protect and restore occupied and adjacent habitat to support threatened species, such as coastal salt marshes and cypress swamps (see Table 2)
- Work with local conservation organizations and land trusts to establish Firefly Sanctuaries in areas with threatened or endemic species or high species diversity
- Work with tourism sites to establish and implement clear guidelines for managers, tour operators, and visitors to ensure that fireflies are protected from tourism-related threats [e.g., 17]
- Mitigate light pollution close to firefly habitat through educational outreach programs, Dark Sky Initiatives, and updates to city lighting ordinances
- Refrain from using pesticides, particularly insecticides and molluscicides, in areas used by fireflies, as these can kill fireflies and their prey, and may have other unintended consequences

Survey and monitor populations

Surveys and monitoring were identified as key conservation actions for all 18 threatened species and all of the D[illegible]pecies. A shortage of survey efforts and population monitoring for the majority of species--due in no small part to a lack of standardized methodology for tracking them, short species activity windows, difficulty in reaching survey sites, difficulty in identification at the species level, the hazards posed by nocturnal fieldwork, and a general lack of funding--severely limits our ability to track firefly populations over time. Baseline inventories to determine species distributions are needed to better understand the conservation status and needs of individual species. In particular, randomized grid surveys over large geographic areas, coupled with targeted surveys for known threatened species, could help reduce survey bias and increase the scope of survey effort[illegible]

While such surveys at the local, state, and federal levels are recommended, successful survey programs may rest on integrating these efforts with large-scale initiatives across wide geographic areas. Community ("citizen") science projects have a long and illustrious history of engaging public interest while benefiting conservation efforts, and many have effectively incorporated web-based tools [e.g., 71] to increase participation and dissemination of data. For many insects, incorporating species-level identifications in such projects can be challenging due to insects' hyper diversity, small size, often abstruse taxonomy, and difficulty of field identification. In the face of recent insect declines, however, community science has the potential to fulfill a critical need in documenting species distributions and population trends [26,72,73]. For fireflies in particular, Firefly Watch [74] has engaged thousands of community scientists across North America since it started in 2008, and additional resources are now available to aid field-based species identifications [28]. Regional projects such as the Western Firefly Project [75] are also filling data gaps. To gather additional occurrence data for species not typically covered by these programs, volunteers who are trained to identify their local threatened and data deficient species could contribute high-quality, geotagged photographs and details of flash pattern behavior, when applicable, to iNaturalist [76].

Given the apparent rarity or limited abundance of some firefly populations, large-scale collecting should be avoided. Lethal sampling is generally not recommended other than for the purposes of collecting voucher specimens to verify species occurrence. When possible, geotagged voucher photos with corresponding habitat and behavior information should be used in lieu of physical vouchers.

Fill data gaps

More than half (53%) of the species assessed were Data Deficient, indicating that more information is needed to assess these species' extinction risks. Data deficient species tended to be

characterized by cryptic life histories, non-flashing communication behavior, or flightless adult females. For example, a large portion of glow-worm species (79%) and diurnal fireflies (68%) are categorized as Data Deficient, as opposed to 38% of flashing species (S4 Table). The comparatively high rate of data deficiency in glow-worm species is likely due in part to the difficulty in detecting these less conspicuous species. Most glow-worm species have flightless females, while the males of most of these species do not produce light to attract mates. Combined with the nocturnal activity period, diminutive body size, and inconspicuous female light signals of most of these species, it is perhaps not surprising that they are often overlooked. This underscores the need for specialized survey protocols and additional research into firefly species that do not use flash signals in courtship, particularly basic life history studies that examine habitat associations and microhabitat needs, larval and adult diets, activity periods, and threats. Details about priority data d[illegible]ient species can be found in S2 Supporting Information.

Engage and educate

Effective science communication can play an important role in conservation, from garnering public support and attracting funding to driving policy changes and promoting informed decision making. For small yet charismatic animals like fireflies, building up communication efforts may lead to increased support for not only fireflies and their habitats, but invertebrate conservation more broadly. In tandem with the conservation actions discussed here, we recommend increasing outreach and education efforts to share new findings and facilitate collaboration. Workshops, social and popular media, fieldtrips, museum exhibits, community events, and bioblitzes can all be effective means for increasing engagement with firefly conservation.

Conclusions

This paper summarizes the first global IUCN Red List assessments for fireflies. While it does not include all described species in the U.S. and Canada, it represents a substantial step forward in understanding extinction risk for North American species. We now have a foundation from which we can work, which spans the setting of conservation priorities to the establishment of a baseline against which future findings can be compared. We hope the results and implications discussed in this paper will catalyze action to study and conserve fireflies, not just in the U.S. and Canada but everywhere fireflies are found.

Acknowledgements

We thank Ron Lyons, Laura Hughes, and Jason Davis for contributing to the Red List assessments; Annie Nguyen and Aubrey Hornor for assisting with data compilation and georeferencing; Clay Meredith for co-facilitating the IUCN Red List training workshop; and the dedicated staff at the Red List Unit, in particular Janet Scott, for publishing the Red List assessments. We are grateful to the many firefly researchers past and present who have enhanced our understanding of North American fireflies, the vast network of community scientists who share their observations with the scientific community, and the many photographers who contributed their photos to the Red List publications and this paper.

Author contributions

Conceptualization: CEF AW SL SJ. Investigation: CEF AW SL JC LF CH CXPH BF. Writing – original draft: CEF AW SL. Writing – review & editing: CEF AW SL JC LF CH CXPH BF SJ.

References

1. Rodrigues ASL, Pilgrim JD, Lamoreux JF, Hoffmann M, Brooks TM. The value of the IUCN Red List for conservation. Trends Ecol Evol. 2006;21: 71–76. doi:10.1016/j.tree.2005.10.010

2. Hoffmann M, Brooks T, da Fonseca G, Gascon C, Hawkins A, James R, et al. Conservation planning and the IUCN Red List. Endang Species Res. 2008;6: 113–125. doi:10.3354/esr00087

3. IUCN. Table 1a: Number of species evaluated in relation to the overall number of described species, and numbers of threatened species by major groups of organisms. In: IUCN Red List of Threatened Species [Internet]. 2021 [cited 4 Mar 2021]. Available: https://www.iucnredlist.org/resources/summary-statistics

4. Slipinski SA, Leschen R a. B, Lawrence JF. Order Coleoptera Linnaeus, 1758. In : Zhang, Z.-Q. (Ed.) Animal biodiversity: An outline of higher-level classification and survey of taxonomic richness. Zootaxa. 2011;3148: 203–208. doi:10.11646/zootaxa.3148.1.39

5. Gerlach J, Samways MJ, Hochkirch A, Seddon M, Cardoso P, Clausnitzer V, et al. Prioritizing non-marine invertebrate taxa for Red Listing. J Insect Conserv. 2014;18: 573–586. doi:10.1007/s10841-014-9660-6

6. Branham MA, Wenzel JW. The origin of photic behavior and the evolution of sexual communication in fireflies (Coleoptera: Lampyridae). Cladistics. 2003;19: 1–22. doi:10.1111/j.1096-0031.2003.tb00404.x

7. Stanger-Hall KF, Lloyd JE, Hillis DM. Phylogeny of North American fireflies (Coleoptera: Lampyridae): Implications for the evolution of light signals. Mol Phylogenet Evol. 2007;45: 33–49. doi:10.1016/j.ympev.2007.05.013

8. Lewis SM, Cratsley CK. Flash signal evolution, mate choice, and predation in fireflies. Annu Rev Entomol. 2008;53: 293–321. doi:10.1146/annurev.ento.53.103106.093346

9. Lloyd JE. Fireflies (Coleoptera: Lampyridae). In: Capinera JL, editor. Encyclopedia of Entomology. Dordrecht: Springer Netherlands; 2008. pp. 1429–1452. doi:10.1007/978-1-4020-6359-6_3811

10. Martin GJ, Stanger-Hall KF, Branham MA, Da Silveira LFL, Lower SE, Hall DW, et al. Higher-level phylogeny and reclassification of Lampyridae (Coleoptera: Elateroidea). Insect Syst Divers. 2019;3. doi:10.1093/isd/ixz024

11. Brasier AR, Tate JE, Habener JF. Optimized use of the firefly luciferase assay as a reporter gene in mammalian cell lines. Biotechniques. 1989;7: 1116–1122.

12. Lloyd JE. Firefly parasites and predators. Coleopt Bull. 1973;27: 91–106.

13. Viviani VR, Rocha MY, Hagen O. Bioluminescent beetles (Coleoptera: Elateroidea: Lampyridae, Phengodidae, Elateridae) in the municipalities of Campinas, Sorocaba-Votorantim and Rio Claro-

Limeira (SP, Brazil): Biodiversity and influence of urban sprawl. Biota Neotrop. 2010;10: 103–116. doi:10.1590/S1676-06032010000200013

14. Lewis SM, Faust L, De Cock R. The dark side of the light show: Predators of fireflies in the Great Smoky Mountains. Psyche. 2012; e634027. doi:10.1155/2012/634027

15. Hagen O, Santos RM, Schlindwein MN, Viviani VR. Artificial night lighting reduces firefly (Coleoptera: Lampyridae) occurrence in Sorocaba, Brazil. Adv Entomol. 2015;3: 24–32. doi:10.4236/ae.2015.31004

16. Lewis SM. Silent sparks: The wondrous world of fireflies. Princeton, NJ: Princeton University Press; 2016.

17. Lewis SM, Thancharoen A, Wong CH, López-Palafox T, Santos PV, Wu C, et al. Firefly tourism: Advancing a global phenomenon toward a brighter future. Conservat Sci and Prac. 2021;e391. doi:10.1111/csp2.391

18. Fu X, Benno Meyer-Rochow V. Larvae of the firefly *Pyrocoelia pectoralis* (Coleoptera: Lampyridae) as possible biological agents to control the land snail *Bradybaena ravida*. Biol Control. 2013;65: 176–183. doi:10.1016/j.biocontrol.2013.02.005

19. Atkins V, Bell D. The status of the glow-worm *Lampyris noctiluca* L. (Coleoptera: Lampyridae) in England. Lampyrid. 2016;4: 20–35.

20. Gardiner T, Didham RK. Glowing, glowing, gone? Monitoring long-term trends in glow-worm numbers in south-east England. Insect Conserv Divers. 2020;13: 162–174. doi:10.1111/icad.12407

21. Khoo V, Nada B, Kirton LG, Chooi-Khim P. Monitoring the population of the firefly *Pteroptyx tener* along the Selangor River, Malaysia for conservation and sustainable ecotourism. Lampyrid. 2009;2: 162–173.

22. Wong C, Yeap CA. Conservation of congregating firefly zones (CFZs) in peninsular Malaysia. Lampyrid. 2012;2: 174–187.

23. Lewis SM, Wong CH, Owens ACS, Fallon C, Jepsen S, Thancharoen A, et al. A global perspective on firefly extinction threats. BioScience. 2020;70: 157–167. doi:10.1093/biosci/biz157

24. Cardoso P, Leather SR. Predicting a global insect apocalypse. Insect Conserv Divers. 2019;12: 263–267. doi:10.1111/icad.12367

25. Montgomery GA, Dunn RR, Fox R, Jongejans E, Leather SR, Saunders ME, et al. Is the insect apocalypse upon us? How to find out. Biol Conserv. 2020;241: 108327. doi:10.1016/j.biocon.2019.108327

26. Wagner DL, Grames EM, Forister ML, Berenbaum MR, Stopak D. Insect decline in the Anthropocene: Death by a thousand cuts. Proc Natl Acad Sci USA. 2021;118: e2023989118. doi:10.1073/pnas.2023989118

27. Branham M. Lampyridae. In: Leschen R a. B, Beutel RG, Lawrence JF, editors. Coleoptera, Beetles Morphology and Systematics (Elateroidea, Bostrichiformia, Cucujiformia partim). Berlin, Germany: Walter de Gruyter; 2010. pp. 141–149. Available: https://doi.org/10.1515/9783110911213

28. Faust L. Fireflies, glow-worms, and lightning bugs: Identification and natural history of the fireflies of the eastern and central United States and Canada. Athens, GA: University of Georgia Press; 2017.

29. Buschman LL. Larval biology and ecology of *Photuris fireflies* (Lampyridae: Coleoptera) in northcentral Florida. J Kans Entomol Soc. 1984;57: 7–16.

30. Lloyd JE. A naturalist's long walk among shadows of North American *Photuris*: Patterns, outlines, silhouettes... echoes. Bridgen Press; 2018.

31. Lloyd JE. On research and entomological education II: A conditional mating strategy and resource-sustained lek(?) in a classroom firefly (Coleoptera: Lampyridae; *Photinus*). Fla Entomol. 1998;81: 261–272. doi:10.2307/3495917

32. Rooney JA, Lewis SM. Notes on the life history and mating behavior of *Ellychnia corrusca* (Coleoptera: Lampyridae). Fla Entomol. 2000; 324–324.

33. Faust L, Faust H. The occurrence and behaviors of North American fireflies (Coleoptera: Lampyridae) on milkweed, *Asclepias syriaca* L. Coleopt Bull. 2014;68: 283–291. doi:10.1649/0010-065X-68.2.283

34. Zaragoza-Caballero S. Lampyridae (Coleoptera). In: García-Aldrete AN, Ayala R, editors. Artrópodos de Chamela. Instituto de Biología, Universidad Nacional Autónoma de México, D.F.; 2004. pp. 139–150.

35. Lloyd JE. Aggressive mimicry in *Photuris*: Firefly femmes fatales. Science. 1965;149: 653–654. doi:10.1126/science.149.3684.653

36. Lloyd JE. Occurrence of aggressive mimicry in fireflies. Fla Entomol. 1984;67: 368–376. doi:10.2307/3494715

37. Faust LF, Forrest TG. Bringing light to the lives of the shadow ghosts, *Phausis inaccensa* (Coleoptera: Lampyridae). Am Entomol. 2017;63: 177–189. doi:10.1093/ae/tmx027

38. Lloyd JE. Chemical communication in fireflies. Environ Entomol. 1972;1: 265–266.

39. Lloyd JE. On research and entomological education VI: Firefly species and lists, old and now. Fla Entomol. 2003; 99–113.

40. Cicero J. Two new genera and one new species of firefly (Coleoptera: Lampyridae: Lampyrinae: Lampyrini), with notes on their biology. Pan-Pac Entomol. 2006;82: 200–207.

41. Heckscher CM. *Photuris mysticalampas* (Coleoptera: Lampyridae): A new firefly from peatland floodplain forests of the Delmarva Peninsula. Entomol News. 2013;123: 93–100. doi:10.3157/021.123.0202

42. Faust LF, Davis J. A new species of *Photuris* Dejean (Coleoptera: Lampyridae) from a Mississippi cypress swamp, with notes on its behavior. Coleopt Bull. 2019;73: 97–113. doi:10.1649/0010-065X-73.1.97

43. Lloyd JE. 62. Lampyridae Latreille 1817. In: Arnett J Ross H, Thomas MC, Skelley PE, Frank JH, editors. American Beetles, Volume II Polyphaga: Scarabaeoidea through Curculionoidea. Boca Raton, FL: CRC Press; 2002. pp. 187–196.

44. ITIS. Integrated Taxonomic Information System (ITIS). 2020 [cited 13 Jan 2020]. Available: https://itis.gov/

45. Cicero J. World Lampyridae, Lycidae, Phengodidae, Drilidae and miscellany. 2013 [cited 29 Mar 2021]. Available: http://mycantharoidea.arizona.edu/

46. IUCN. IUCN Red List categories and criteria, version 3.1, second edition. In: IUCN [Internet]. 2012 [cited 29 Mar 2021]. Available: https://www.iucn.org/content/iucn-red-list-categories-and-criteria-version-31-second-edition

47. IUCN Standards and Petitions Committee. Guidelines for using the IUCN Red List Categories and Criteria. Version 14. Prepared by the Standards and Petitions Committee. 2019. Available: http://www.iucnredlist.org/documents/RedListGuidelines.pdf

48. IUCN. The IUCN Red List of Threatened Species. In: IUCN Red List of Threatened Species [Internet]. 2021 [cited 29 Mar 2021]. Available: https://www.iucnredlist.org/en

49. Environmental Protection Agency. Ecoregions of North America. 2018 [cited 27 May 2021]. Available: https://data.nal.usda.gov/dataset/ecoregions-north-america

50. Hoffmann M, Hilton-Taylor C, Angulo A, Böhm M, Brooks TM, Butchart SHM, et al. The impact of conservation on the status of the world’s vertebrates. Science. 2010;330: 1503–1509. doi:10.1126/science.1194442

51. Richman NI, Böhm M, Adams SB, Alvarez F, Bergey EA, Bunn JJS, et al. Multiple drivers of decline in the global status of freshwater crayfish (Decapoda: Astacidea). Phil Trans R Soc B. 2015;370: 20140060. doi:10.1098/rstb.2014.0060

52. Payne JL, Finnegan S. The effect of geographic range on extinction risk during background and mass extinction. PNAS. 2007;104: 10506–10511. doi:10.1073/pnas.0701257104

53. Chichorro F, Juslén A, Cardoso P. A review of the relation between species traits and extinction risk. Biol Conserv. 2019;237: 220–229. doi:10.1016/j.biocon.2019.07.001

54. Nolte D, Boutaud E, Kotze DJ, Schuldt A, Assmann T. Habitat specialization, distribution range size and body size drive extinction risk in carabid beetles. Biodivers Conserv. 2019;28: 1267–1283. doi:10.1007/s10531-019-01724-9

55. Reed JM, Nguyen A, Owens ACS, Lewis SM. Linking the seven forms of rarity to extinction threats and risk factors: an assessment of North American fireflies. Biodivers Conserv. 2020;29: 57–75. doi:10.1007/s10531-019-01869-7

56. Lloyd JE. Observations on the biology of three luminescent beetles (Coleoptera: Lampyridae, Elateridae). Ann Entomol Soc Am. 1965;58: 588–591. doi:10.1093/aesa/58.4.588

57. Williams AP, Cook ER, Smerdon JE, Cook BI, Abatzoglou JT, Bolles K, et al. Large contribution from anthropogenic warming to an emerging North American megadrought. Science. 2020;368: 314–318. doi:10.1126/science.aaz9600

58. Konikow LF. Groundwater depletion in the United States (1900–2008): USGS Scientific Investigations Report 2013–5079. 2013 p. 63. Available: https://pubs.usgs.gov/sir/2013/5079/

59. Wu W-Y, Lo M-H, Wada Y, Famiglietti JS, Reager JT, Yeh PJ-F, et al. Divergent effects of climate change on future groundwater availability in key mid-latitude aquifers. Nat Commun. 2020;11: 3710. doi:10.1038/s41467-020-17581-y

60. Zektser S, Loáiciga HA, Wolf JT. Environmental impacts of groundwater overdraft: selected case studies in the southwestern United States. Env Geol. 2005;47: 396–404. doi:10.1007/s00254-004-1164-3

61. Buschman L. Field guide to western North American fireflies. 2016 p. 33. Available: http://entomology.k-state.edu/doc/WesternFireflies%20March%202016a.pdf

62. Dahl TE. Wetlands losses in the United States, 1780's to 1980's. Report to the Congress. National Wetlands Inventory, St. Petersburg, FL (USA); 1990 Jan. Report No.: PB-91-169284/XAB. Available: https://www.osti.gov/biblio/5527872-wetlands-losses-united-states-report-congress

63. Dahl TE. Status and trends of wetlands in the conterminous United States 2004 to 2009. U.S. Department of the Interior, U.S. Fish and Wildlife Service, Fisheries and Habitat Conservation; 2011.

64. Sucik MT, Marks E. The status and recent trends of wetlands in the United States. Ames, IA: USDA Natural Resources Conservation Service; 2014 p. 25.

65. Vaz S, Guerrazzi MC, Rocha M, Faust L, Khattar G, Mermudes J, et al. On the intertidal firefly genus *Micronaspis* Green, 1948, with a new species and a phylogeny of Cratomorphini based on adult and larval traits (Coleoptera: Lampyridae). Zool Anz. 2021;292: 64–91. doi:10.1016/j.jcz.2021.01.002

66. Owens ACS, Cochard P, Durrant J, Farnworth B, Perkin EK, Seymoure B. Light pollution is a driver of insect declines. Biol Conserv. 2020;241: 108259. doi:10.1016/j.biocon.2019.108259

67. Owens ACS, Lewis SM. The impact of artificial light at night on nocturnal insects: A review and synthesis. Ecol Evol. 2018;8: 11337–11358. doi:10.1002/ece3.4557

68. Owens ACS, Lewis SM. Narrow-spectrum artificial light silences female fireflies (Coleoptera: Lampyridae). Insect Conserv Divers. 2021;14: 199–210. doi:10.1111/icad.12487

69. Owens ACS, Lewis SM. Effects of artificial light on growth, development, and dispersal of two North American fireflies (Coleoptera: Lampyridae). J Insect Physiol. 2021;130: 104200. doi:10.1016/j.jinsphys.2021.104200

70. USFWS. Environmental Conservation Online System (ECOS). Online database. In: Environmental Conservation Online System (ECOS) [Internet]. 2021 [cited 29 Mar 2021]. Available: https://ecos.fws.gov/ecp0/reports/ad-hoc-species-report-input

71. Sullivan BL, Wood CL, Iliff MJ, Bonney RE, Fink D, Kelling S. eBird: A citizen-based bird observation network in the biological sciences. Biol Conserv. 2009;142: 2282–2292. doi:10.1016/j.biocon.2009.05.006

72. Harvey JA, Heinen R, Armbrecht I, Basset Y, Baxter-Gilbert JH, Bezemer TM, et al. International scientists formulate a roadmap for insect conservation and recovery. Nat Ecol Evol. 2020;4: 174–176. doi:10.1038/s41559-019-1079-8

73. Kawahara AY, Reeves LE, Barber JR, Black SH. Opinion: Eight simple actions that individuals can take to save insects from global declines. Proc Natl Acad Sci USA. 2021;118: e2002547117. doi:10.1073/pnas.2002547117

74. Firefly Watch Community Science Project. In: Mass Audubon [Internet]. [cited 25 May 2021]. Available: https://www.massaudubon.org/get-involved/community-science/firefly-watch

75. Western Firefly Project: A Community Science Initiative. In: Natural History Museum of Utah [Internet]. 25 Jul 2017 [cited 25 May 2021]. Available: https://nhmu.utah.edu/citizen-science/fireflies

76. iNaturalist. In: iNaturalist [Internet]. [cited 25 May 2021]. Available: https://www.inaturalist.org/

Supporting information

S1 Table. Firefly species distributions in the U.S. and Canada. Species name: †: Awaiting publication on the IUCN Red List. RL category: DD*: Potentially threatened DD species. Occurrence and Distribution: Extant (E): Species has been reported since 2000; Presence uncertain (U): Species reported prior to 2000; Possibly Extant (PE): No known records but habitat or locality is appropriate and species may occur here; Possibly Extinct (PX): Species has not been seen in many years despite comprehensive survey efforts.

S2 Table. Firefly taxonomic experts consulted in this project. Contributor: Contributed information to Red List assessments; Assessor: Co-authored Red List assessments; Reviewer: Reviewed Red List assessments; Determiner: Verified species IDs.

S3 Table. Recommended conservation actions for threatened firefly species in the U.S. and Canada.

S4 Table. North American firefly genera with total number of species assessed, percentage of threatened and data deficient species, and behavioral type.

S5 Table. Potentially threatened DD firefly species.

Fig 1A

Click here to access/download;Figure;Fig 1A.tif

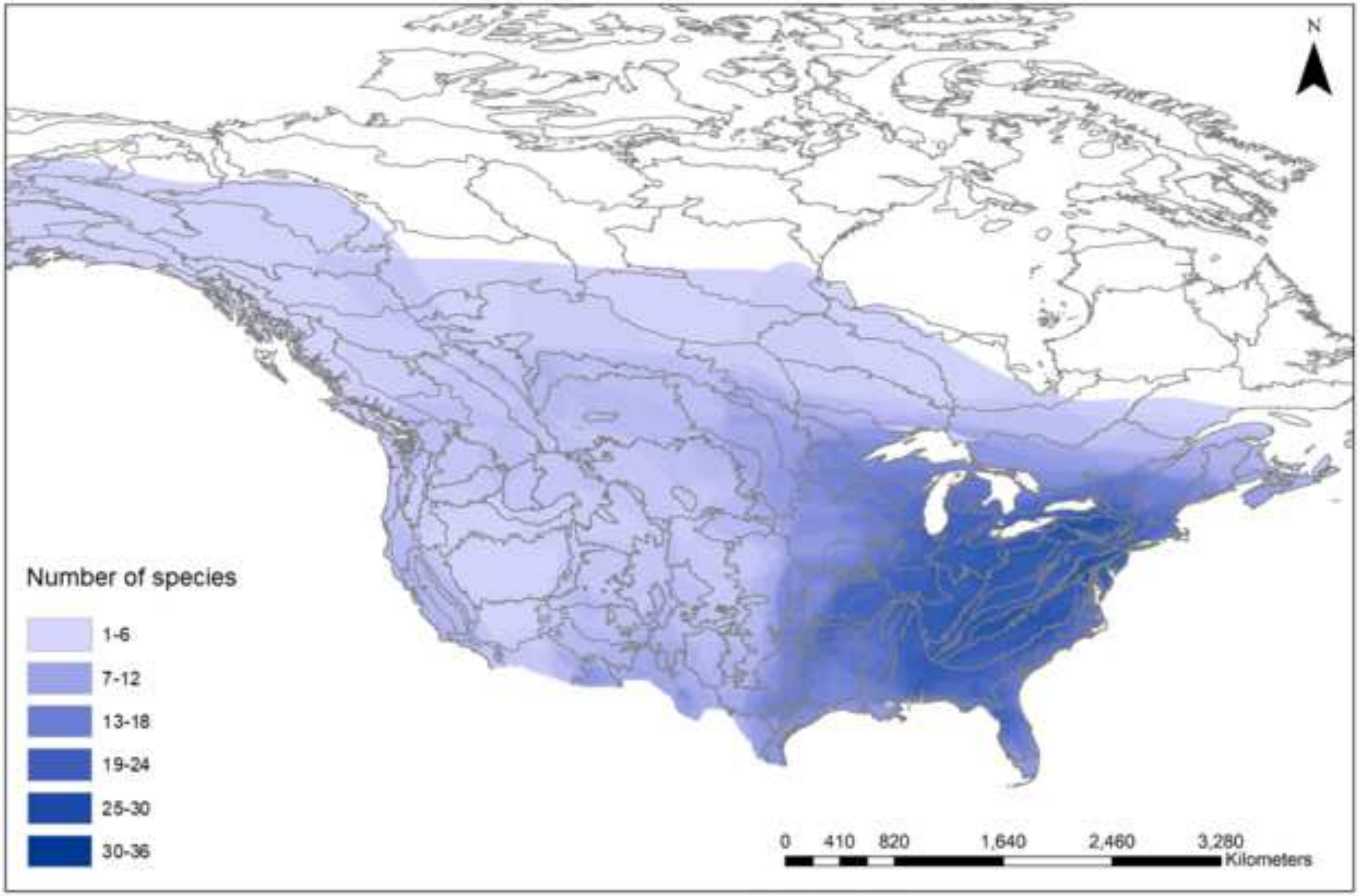

Fig 1B

Click here to access/download;Figure;Fig 1B.tif

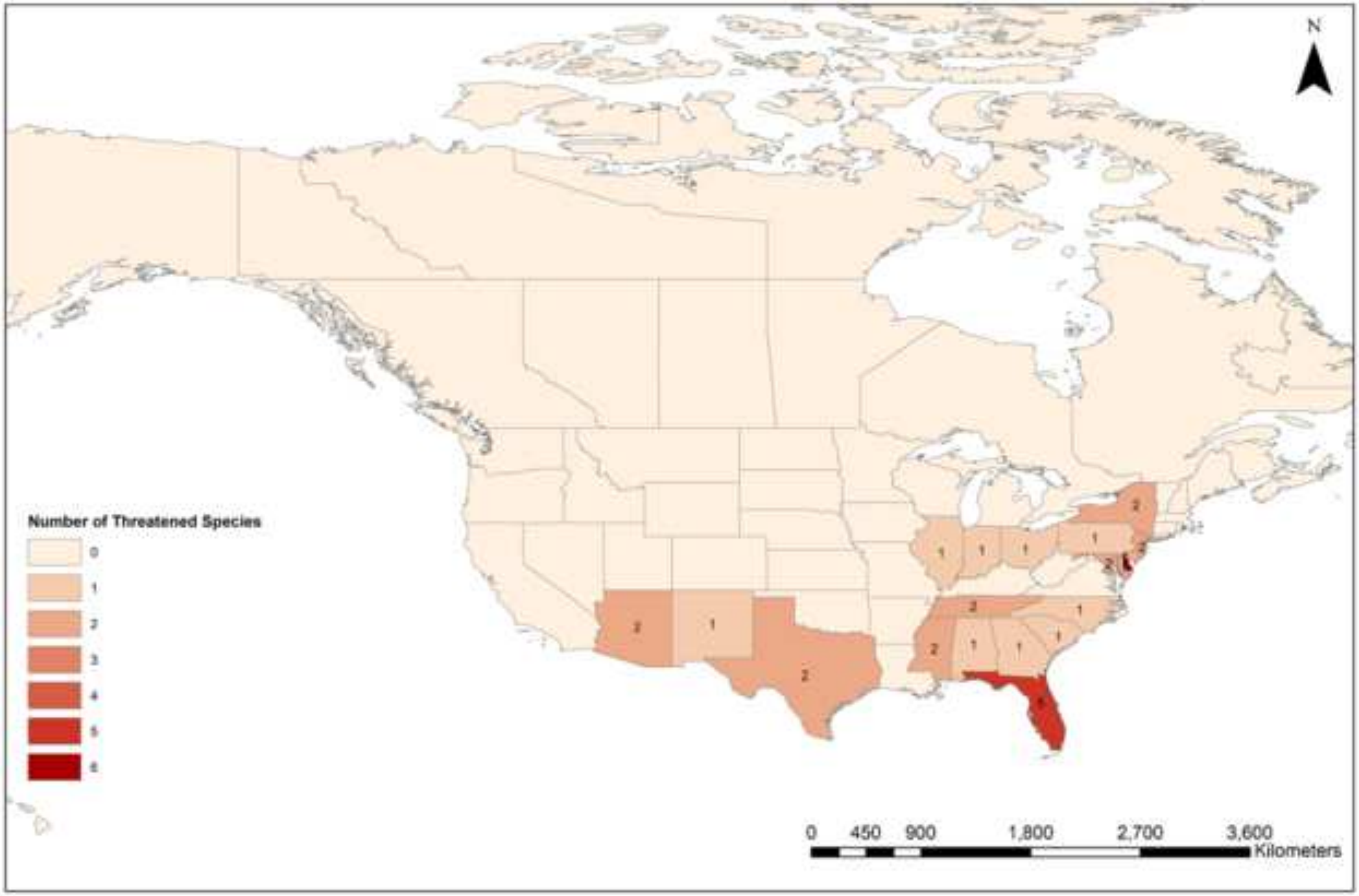

Fig 1C

Click here to access/download;Figure;Fig 1C.tif

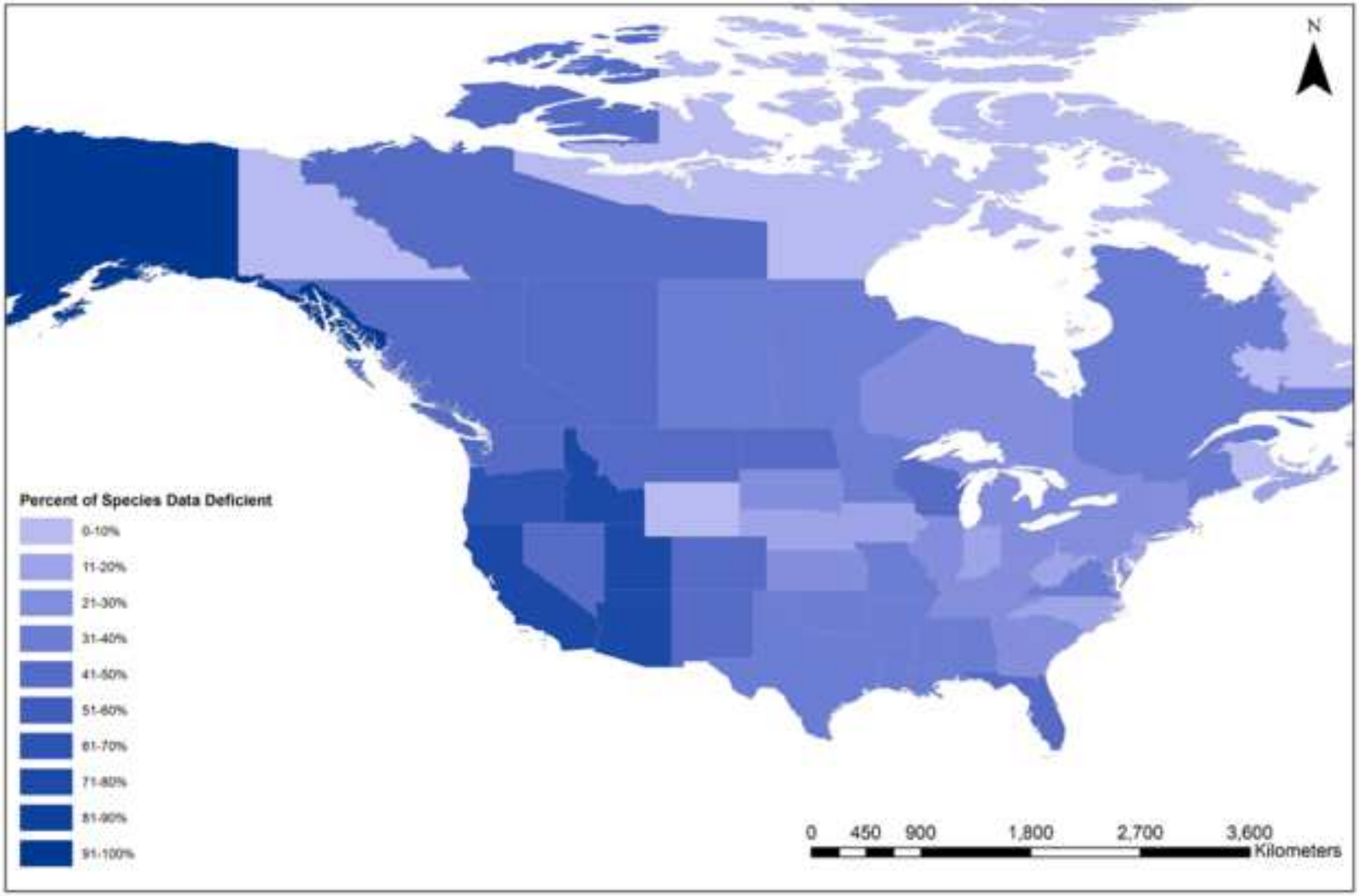

Fig 2

Click here to access/download;Figure;Fig 2.tif

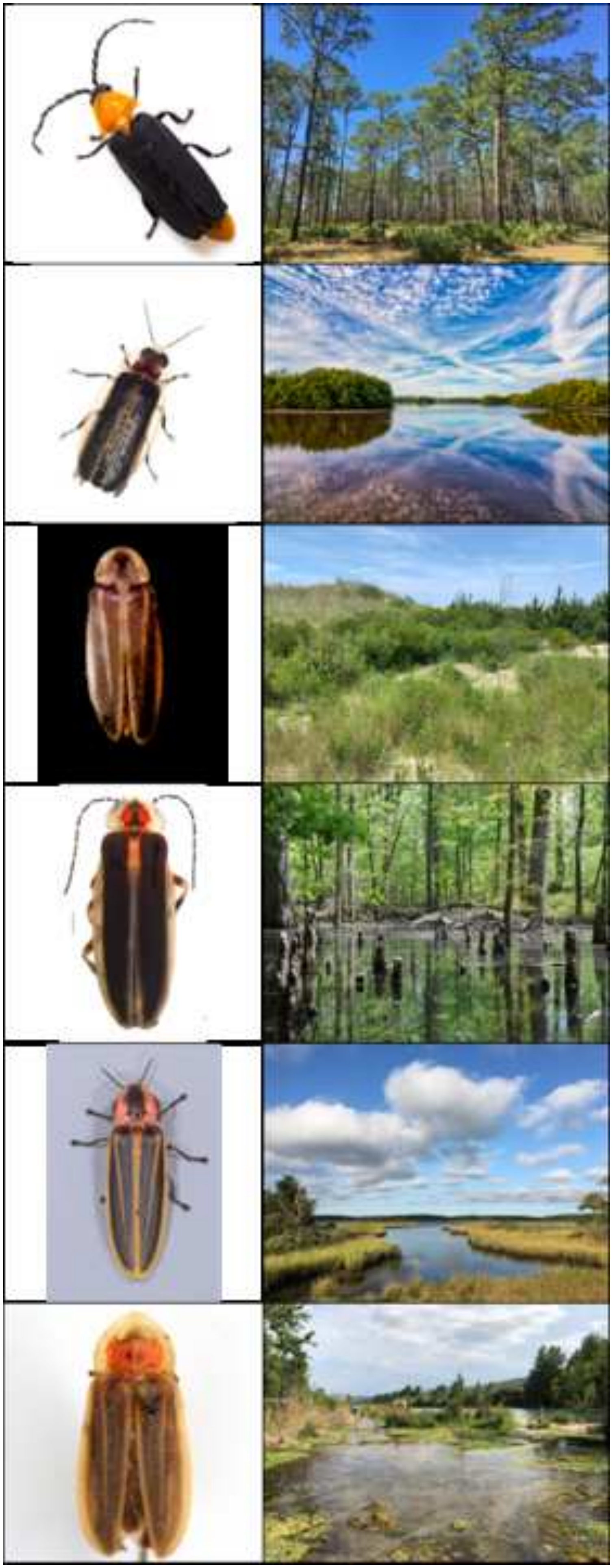

Click here to access/download
Supporting Information - Compressed/ZIP File Archive
Supporting Information.zip
